# Effects of powered versus passive-elastic ankle foot prostheses on leg muscle activity during level, uphill and downhill walking

**DOI:** 10.1098/rsos.220651

**Published:** 2022-12-14

**Authors:** Zane A. Colvin, Jana R. Montgomery, Alena M. Grabowski

**Affiliations:** ^1^ Applied Biomechanics Lab, Department of Integrative Physiology, University of Colorado Boulder, Boulder, CO, USA; ^2^ VA Eastern Colorado Healthcare System, Denver, CO, USA

**Keywords:** electromyography, prosthesis, slope, timing, transtibial, amputation

## Abstract

People with transtibial amputation (TTA) using passive-elastic prostheses have greater leg muscle activity and metabolic cost during level-ground and sloped walking than non-amputees. Use of a stance-phase powered (BiOM) versus passive-elastic prosthesis reduces metabolic cost for people with TTA during level-ground, +3° and +6° walking. Metabolic cost is associated with muscle activity, which may provide insight into differences between prostheses. We measured affected leg (AL) and unaffected leg (UL) muscle activity from ten people with TTA (6 males, 4 females) walking at 1.25 m s^−1^ on a dual-belt force-measuring treadmill at 0°, ±3°, ±6° and ±9° using their own passive-elastic and the BiOM prosthesis. We compared stride average integrated EMG (iEMG), peak EMG and muscle activity burst duration. Use of the BiOM increased UL lateral gastrocnemius iEMG on downhill slopes and AL biceps femoris on +6° and +9° slopes, and decreased UL rectus femoris on uphill slopes, UL vastus lateralis on +6° and +9°, and soleus and tibialis anterior on a +9° slope compared to a passive-elastic prosthesis. Differences in leg muscle activity for people with TTA using a passive-elastic versus stance-phase powered prosthesis do not clearly explain differences in metabolic cost during walking on level ground and slopes.

## 1. Introduction

People with a transtibial amputation (TTA) who use a passive-elastic prosthesis exhibit increased leg muscle activity in both legs during walking on level ground compared to non-amputees [1–3]. This likely results from biomechanical differences between legs due in part to the difference in mechanical function provided by a passive-elastic prosthesis compared to a biological foot and ankle joint. Passive-elastic prostheses are typically comprised of carbon fibre and attach via a metal pylon beneath a rigid socket that encompasses the residual limb. A passive-elastic prosthesis functions as a mechanical spring that stores and returns elastic energy during the stance phase of walking. Because of this spring-like function, such prostheses are often referred to as energy storage and return (ESAR) prostheses. During level-ground walking at 1.5 m s^−1^ in people with TTA, an ESAR prosthesis releases less than one-half the mechanical energy and less than one-eighth the peak mechanical power normally generated by the soleus and gastrocnemius [4], muscles that span the ankle joint and are primarily responsible for propulsion and ankle joint plantar-flexion. ESAR prostheses cannot generate net positive work or power for prosthetic ankle plantar-flexion nor dorsi-flex in a manner comparable to a biological ankle during walking.

To compensate for the loss of function provided by an ESAR prosthesis compared to a biological foot and ankle, people with TTA change leg muscle activity in their affected leg (AL) and unaffected leg (UL) to walk on level ground at a steady speed compared to non-amputees [1,2]. People with TTA using an ESAR prosthesis increase the magnitude of AL hip extensor muscle activity during early stance to maintain preferred steady-speed level-ground walking compared to non-amputees [1]. People with TTA using an ESAR prosthesis also increase the magnitude of AL knee flexor muscle activity during early to mid-stance and have greater knee flexor and extensor co-activation compared to non-amputees during level-ground walking [1]. In addition, people with TTA using ESAR prostheses increase the magnitude of UL gluteus medius activity compared to non-amputees during mid- to late-stance during level-ground walking at 1.5 m s^−1^ [3]. An increase in the magnitude of muscle activity is associated with increased muscle force generation needed to produce mechanical work and power during walking. To produce force, muscles use oxidative metabolism, which incurs a metabolic cost. Thus, an increased magnitude of muscle activity in people with TTA using an ESAR prosthesis is likely associated with greater muscle force development and thus an increased metabolic cost compared to non-amputees.

The increased magnitude of muscle activity in people with TTA compared to non-amputees could be mitigated with the use of a stance-phase powered ankle–foot prosthesis (BiOM, Ottobock, Duderstadt, Germany). The BiOM (now called the Empower) is the only commercially available ankle–foot prosthesis that provides stance-phase powered prosthetic ankle plantar-flexion during walking through use of a battery and series elastic actuator, which includes a motor and in-series spring [5]. Use of the BiOM battery-powered prosthesis contributes 57–63% more push-off work in the trailing AL during the step-to-step transition compared to use of an ESAR prosthesis in people with TTA walking at 0.75–1.75 m s^−1^ on level ground [6,7]. The increased mechanical work and power provided by the BiOM prosthesis suggests that a lower magnitude of muscle activity and metabolic power may be required by people with TTA compared to using an ESAR prosthesis. Compared to an ESAR prosthesis, use of the BiOM powered ankle–foot prosthesis resulted in 7–20% lower metabolic power during level-ground walking at 0.75–1.75 m s^−1^ [6,7], and 5% lower metabolic power during walking at 1.25 m s^−1^ on +3° and +6° uphill slopes [8]. However, Montgomery & Grabowski [8] found no significant differences in metabolic power when people with TTA used the BiOM compared to their own ESAR prosthesis during downhill walking on slopes of −3°, −6° and −9° at 1.25 m s^−1^. These results suggest that use of the BiOM changes the magnitude of AL and UL muscle activity in people with TTA compared to use of an ESAR prosthesis and that these changes may depend on slope.

When people with TTA used the BiOM compared to an ESAR prosthesis to walk on level ground at a normalized speed, they increased muscle activation, measured by integrated electromyography (iEMG) over a stride, in their UL gluteus medius and AL vastus medialis [9]. However, these iEMG results do not corroborate previous findings that metabolic power is lower in people with TTA using the BiOM compared to ESAR prosthesis during level-ground walking [6]. Moreover, the muscle activity elicited by people with TTA using the BiOM compared to an ESAR prosthesis to walk on uphill and downhill slopes is unknown. Non-amputees have greater muscle activity amplitude and duration of their hip extensors, knee extensors and amplitude of their ankle plantar-flexors during ground contact when walking at a self-selected speed on a +39% grade (+21.3°) compared to level ground [10]. Non-amputees also have greater muscle activity amplitude and duration of their knee extensors and amplitude of their ankle dorsiflexors when walking at a self-selected speed on a −39% grade (−21.3°) compared to level ground [10]. The increased muscle activity of the hip and knee extensors and ankle plantar-flexors on uphill slopes is likely a response to the increased positive work required to raise the centre of mass (COM), whereas the increased muscle activity of the knee extensors on downhill slopes is likely a response to the greater magnitude of negative work required to lower the COM. Although walking uphill requires greater mechanical work and extensor muscle activity magnitude compared to level-ground walking, the prosthetic ankle plantar-flexion power provided by the BiOM prosthesis could mitigate this increased muscle activity magnitude for people with TTA to walk uphill.

Previous studies have compared muscle activity of people with TTA and non-amputees by determining the average muscle activity magnitude from surface electromyography (EMG) over a pre-determined time period [1,3]. Integrated EMG (iEMG) is calculated by integrating muscle activity over time and provides a measure of overall muscle activation. However, iEMG does not describe the peak amplitude or muscle activity burst duration. Thus, we determined iEMG, peak EMG magnitude and muscle activity duration of the leg muscles for the AL and UL of people with TTA using their own ESAR prosthesis and the BiOM powered ankle–foot prosthesis during level, uphill and downhill walking. Because use of a powered prosthesis provides net positive plantar-flexion work and power during walking and decreases metabolic power compared to use of an ESAR prosthesis during level-ground and uphill walking, we hypothesized that: (i) iEMG and peak EMG magnitude of the hip extensors and knee extensors of each leg would decrease and muscle activity burst duration would not differ during uphill and level-ground walking when subjects with TTA used the BiOM compared with an ESAR prosthesis and (ii) iEMG, peak EMG magnitude and muscle activity burst duration of all leg muscles for the AL and UL would not differ during downhill walking when subjects with TTA used the BiOM compared with an ESAR prosthesis. The differences in leg muscle activity between these prosthetic designs and on a range of slopes during walking will inform future powered prosthetic designs that could indirectly reduce metabolic cost.

## 2. Methods

### 2.1. Subject recruitment

Ten adults (mean ± s.d.: age 42 ± 11 years, mass without a prosthesis 77.3 ± 14.8 kg) with a TTA participated (table 1). All subjects self-reported that they were at or above a K3 Medicare Functional Classification Level and free of neurological, cardiovascular or musculoskeletal disease or injury other than that associated with an amputation. All subjects gave written informed consent prior to participating according to a protocol approved by the United States Department of Veteran Affairs' Human Subjects Institutional Review Board (COMIRB no. 12-0553) and in accordance with the principles expressed in the Declaration of Helsinki.

**Table 1.** Subject anthropometric data and their own elastic storage and return (ESAR) prosthetic foot model.

sex	height (m)	mass with BiOM (kg)	mass with ESAR (kg)	ESAR prosthetic foot model
F	1.66	59.5	58.0	Freedom Innovations Renegade
F	1.66	65.3	61.7	Ottobock Triton IC60
F	1.68	69.4	68.5	Össur Pro-flex XC
M	1.75	72.1	70.3	Freedom Innovations Renegade
M	1.71	78.0	77.0	Össur Vari-flex
F	1.71	84.1	81.8	Össur Vari-flex XC
M	1.82	89.4	88.9	College Park Soleus
M	1.85	96.2	95.3	Össur Proflex
M	1.83	97.1	95.5	Ability Dynamics Rush 81
M	1.82	102.3	100.2	Ability Dynamics Rush 87
*average (s.d.)*	*1.70 (0.08)*	*81.3 (14.7)*	*79.7 (15.0)*	

### 2.2. Experimental protocol

The present data were collected as part of a larger study that included measurements of metabolic rates [8], data not reported in the present study. The overall experimental protocol occurred over five sessions. These sessions included a tuning session, three sessions where we measured metabolic rates and a final session where we measured biomechanics and surface electromyography (sEMG). During the tuning session, subjects were aligned and tuned with the BiOM prosthesis (BiOM T2, Ottobock, Duderstadt, Germany) while they walked at 1.25 m s^−1^ on slopes of 0°, ±3°, ±6° and ±9°. During the second, third and fourth sessions, we collected metabolic rates while subjects walked at 1.25 m s^−1^ on slopes of 0°, ±3°, ±6° and ±9° using their own ESAR prosthesis and the BiOM prosthesis, a total of 14 5-min trials that were randomized. During the fifth session, we collected kinetic, kinematic and sEMG data while subjects walked at 1.25 m s^−1^ on slopes of 0°, ±3°, ±6° and ±9° using their own ESAR prosthesis and the BiOM prosthesis, a total of 14 approximately 1-min trials that were randomized.

During the first session, subjects were aligned to the BiOM prosthesis by a certified prosthetist and then we placed reflective markers bilaterally on subjects’ legs and hips over joint centres and placed clusters of markers on lower limb segments [8]. For the BiOM prosthesis, we placed reflective markers at the approximate locations of the first and fifth metatarsal heads, and posterior calcaneus based on locations for the UL. Malleoli markers were placed on the encoder, which coincided with the centre of rotation of the BiOM prosthesis in the sagittal plane. Subjects used the BiOM and walked on a dual-belt force-measuring treadmill (Bertec, Columbus, OH, USA) at 1.25 m s^−1^ on 0°, ±3°, ±6° and ±9° slopes while we measured marker trajectories at 100 Hz and ground reaction forces (GRFs) at 1000 Hz (Vicon Industries, Centennial, CO, USA). We used the tablet provided by the manufacturer to iteratively tune the BiOM for each slope while each subject walked on the treadmill until their prosthetic ankle range of motion, peak power, peak moment and net mechanical work normalized to body mass including the prosthesis matched the corresponding biological ankle data of non-amputees and the subject's UL within 2 s.d. of the mean [8].

During the fifth session, subjects walked on the dual-belt force-measuring treadmill at 1.25 m s^−1^ for approximately 1–2 min per slope using their own ESAR prosthesis (table 1) and the BiOM prosthesis tuned with the settings from the first session. During each trial we simultaneously measured GRFs and sEMG at 1000 Hz (Noraxon, Scottsdale, AZ, USA). Subjects were given at least 2 min rest between each of 14 total trials and the trial order was randomized. Prior to sEMG electrode placement, we shaved, cleaned and lightly abraded the skin over 13 leg muscles. Then, we placed self-adhering Ag–AgCl electrodes with a 2 cm inter-electrode distance on the muscles of interest using visualization and palpation to locate the muscle belly [11]. We measured sEMG bilaterally from the biceps femoris long head (BF), gluteus maximus (Gmax), gluteus medius (Gmed), vastus lateralis (VL) and rectus femoris (RF). We also measured sEMG from the UL lateral gastrocnemius (LG), soleus (Sol) and tibialis anterior (TA). We selected muscles to include each major muscle group in each leg.

### 2.3. Data analysis

We analysed GRFs perpendicular to the treadmill surface and sEMG data using custom Matlab scripts (The Mathworks Inc., Natick, MA, USA). We filtered GRF data using a fourth order Butterworth low-pass filter with a 15 Hz cut-off. We detected ground contact using a 20 N perpendicular GRF threshold and determined a stride from initial ground contact of the AL or UL to the subsequent ground contact of the same leg. We excluded strides that included crossover steps (when the foot contacted both force plates during a step). After exclusion of crossover steps, up to 10 (minimum of 5) remaining strides of each trial were then used for sEMG analyses.

First, sEMG data were demeaned, band-pass filtered from 10 to 499 Hz using a fourth order Butterworth filter, rectified, and then low-pass filtered using a fourth order Butterworth filter with a 10 Hz cut-off to create a linear envelope. Then, using a custom Matlab code, we identified the peak sEMG voltage of each stride. We then normalized sEMG voltage over a stride for each muscle to each subject's respective 5–10 stride average peak sEMG voltage [12] when they walked using their ESAR prosthesis on level ground.

We calculated each subject's average iEMG by integrating normalized sEMG voltage with respect to time and removed outliers, defined as an iEMG value greater than 3 absolute deviations from the median for a given muscle of each subject and trial [13,14]. Then, we calculated the average and s.e.m. of stride iEMG for each muscle and each trial from all subjects.

We calculated each subject's average normalized peak EMG over a stride by identifying the maximum normalized sEMG voltage from 5 to 10 strides and excluded outliers that were greater than 3 absolute deviations from the median for a given muscle of each subject and trial. Then, we calculated the average and s.e.m. of stride peak EMG for each muscle and each trial from all subjects.

We identified the primary burst of activity for each muscle of each subject and trial using visual inspection from 5 to 10 strides [15]. We calculated average muscle activity onset and offset of the primary burst of activity for each subject and trial by averaging onsets and offsets as a percentage of a stride relative to the initial contact of the respective leg. Onsets and offsets were identified when the normalized sEMG voltage crossed a threshold that was 3 s.d. greater than the average inactive period. The inactive period was defined as the average normalized sEMG value over at least 25 ms of the lowest normalized value during 5–10 strides [16,17]. We then eliminated outliers that were greater than 3 absolute deviation from the median onset or offset for a given muscle of each subject and trial. Lastly, we calculated the average and s.e.m. of muscle activity burst onset and offset as a percentage of a stride for each muscle and each trial from all subjects.

### 2.4. Statistics

We constructed linear mixed effects models to determine whether prosthesis type (ESAR and BiOM) or slope had a main effect or interaction effect on stride iEMG, peak EMG magnitude and the duration of the primary burst of muscle activity of each muscle. We fitted a separate model for uphill slopes and level ground, and downhill slopes and level ground for each variable and each muscle of each leg. The fixed effects in our models were prosthesis type (categorical) and slope (continuous) with subject as a random effect. Interaction effects between prosthesis type and slope were included in our models but were dropped from the model if we did not detect significance (*p* < 0.05). In each model, if we detected a significant main effect of prosthesis or slope, the unstandardized fixed effect (*B*) is reported as the change in the dependent variable (iEMG, peak EMG or burst duration) value (dependent variable = *B* × independent variable + intercept). However, if no significance was detected, results of the model were not reported. Because prosthesis type is a categorical variable in the regression, the value of *B* (relative to an effect seen in prosthesis type) is the change in predicted value associated with use of the BiOM. If significant interaction effects were present in any model, *B* values were not reported and pairwise comparisons of estimated marginal means were made to determine where significant differences occurred. Significance was determined with *α* = 0.05 and when multiple comparisons were present, a Bonferroni correction was applied. All statistical analyses were performed using the nlme and emmeans packages in R-Studio [18,19].

## 3. Results

### 3.1. Unaffected leg integrated electromyography

**Figure 1.**
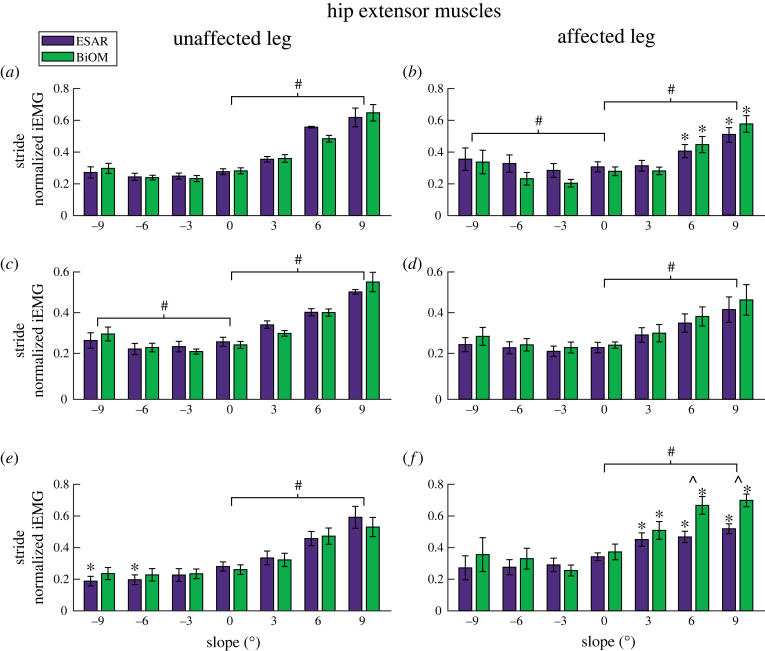
Average (±s.e.m) normalized integrated EMG (iEMG) over a stride for the unaffected leg and affected leg gluteus maximus (*a*,*b*), gluteus medius (*c*,*d*) and biceps femoris (*e*,*f*) muscles while subjects walked at 1.25 m s^−1^ using their own elastic storage and return (ESAR—purple) prosthesis and the BiOM (green) powered prosthesis on slopes of 0°, ±3°, ±6° and ±9°. Hash symbol (#) indicates a significant main effect of slope. Asterisk (*) indicates a significant pairwise comparison difference from 0° using the same prosthesis. Hat symbol (^) indicates a significant pairwise comparison difference between prosthesis type on a specific slope. *α* = 0.05.

For the UL hip extensors, Gmax and Gmed iEMG increased for each steeper uphill slope (*B* = 0.03–0.04 per 1° increment, *p* < 0.001, figure 1) and Gmed iEMG increased with each steeper downhill slope (*B* = −0.004 per 1° increment, *p* = 0.031, figure 1*c*). BF iEMG increased with each steeper uphill slope and decreased with each steeper downhill slope (*B* = 0.009–0.03 per 1° increment, *p* < 0.001, figure 1*e*). We also found an interaction effect (*p* = 0.025), where compared to level ground, BF iEMG was lower on −9° and −6° when using the ESAR prosthesis (*p* = 0.003–0.005) but did not change when using the BiOM prosthesis.

**Figure 2.**
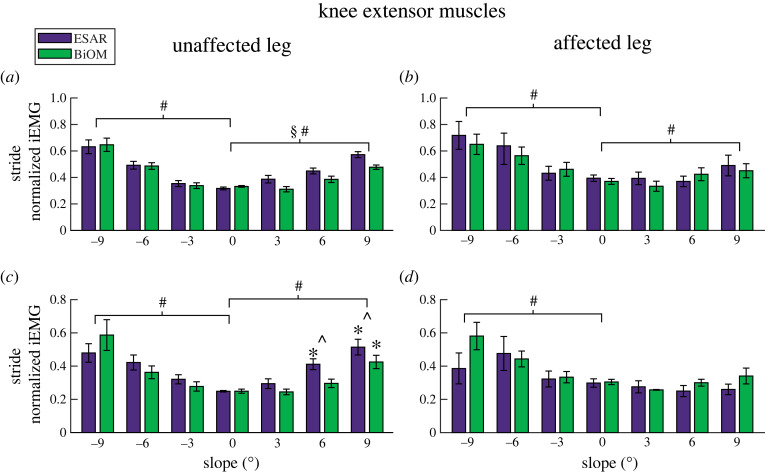
Average (±s.e.m.) normalized integrated EMG (iEMG) over a stride of the unaffected leg and affected leg rectus femoris (*a*,*b*) and vastus lateralis (*c*,*d*) muscles while subjects walked using their own elastic storage and return (ESAR—purple) prosthesis and the BiOM (green) powered prosthesis on slopes of 0°, ±3°, ±6° and ±9°. Hash symbol (#) indicates a significant main effect of slope. Section symbol (§) indicates a significant main effect of prosthesis type. Asterisk (*) indicates a significant pairwise comparison difference from 0° using the same prosthesis. Hat symbol (^) indicates a significant pairwise comparison difference between prosthesis type on a specific slope. *α* = 0.05.

For the UL knee extensors, RF iEMG increased for each steeper uphill (*B* = 0.021 per 1° increment, *p* < 0.0001, figure 2*a*) and downhill (*B* = −0.035 per 1° increment, *p* < 0.001, figure 2*a*) slope and was lower when using the BiOM compared to the ESAR prosthesis on uphill slopes (*B* = −0.04, *p* < 0.001, figure 2). VL iEMG increased for each steeper uphill (*B* = 0.031 per 1° increment, *p* < 0.001, figure 2*c*) and downhill (*B* = −0.032 per 1° increment, *p* < 0.001, figure 2*c*) slope. We also found an interaction effect (*p* = 0.025, figure 2*c*), where compared to level ground, VL iEMG increased on +6° while using the ESAR prosthesis (*p* < 0.001), increased on +9° regardless of prosthesis, (*p* < 0.001) and use of the BiOM compared to the ESAR prosthesis lowered VL iEMG on +6° and +9° (*p* = 0.004–0.032).

**Figure 3.**
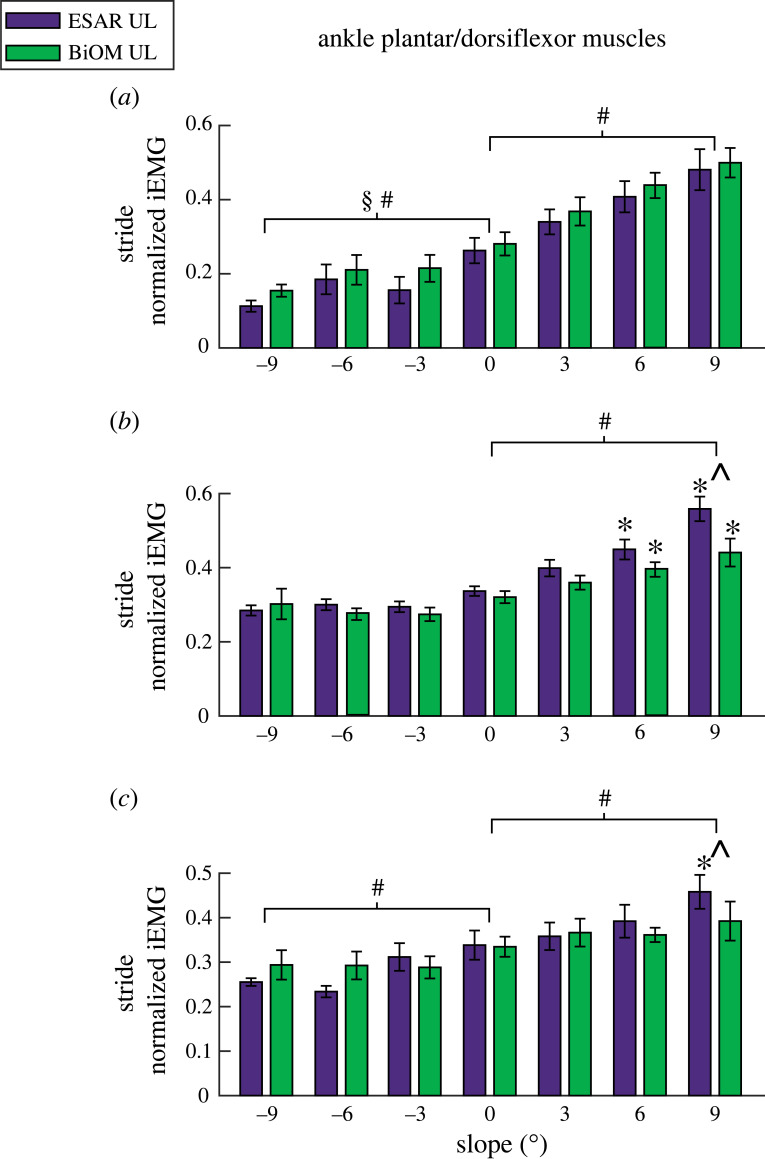
Average (±s.e.m.) normalized integrated EMG (iEMG) over a stride of the unaffected leg (UL) lateral gastrocnemius (*a*), soleus (*b*) and tibialis anterior (*c*) muscles while subjects walked using their own elastic storage and return (ESAR—purple) prosthesis and the BiOM (green) powered prosthesis on slopes of 0°, ±3°, ±6° and ±9°. Hash symbol (#) indicates a significant main effect of slope. Section symbol (§) indicates a significant main effect of prosthesis type. Asterisk (*) indicates a significant pairwise comparison difference from 0° using the same prosthesis. Hat symbol (^) indicates a significant pairwise comparison difference between prosthesis type on a specific slope. *α* = 0.05.

For the UL ankle plantar- and dorsiflexors, LG iEMG increased for each steeper uphill slope (*B* = 0.024 per 1° increment, *p* < 0.001, figure 3*a*), decreased for each steeper downhill slope (*B* = 0.009 per 1° increment, *p* < 0.001, figure 3*a*) and was higher when using the BiOM compared to the ESAR prosthesis on downhill slopes (*B* = 0.02, *p* = 0.024, figure 3*a*). Sol iEMG increased for each steeper uphill slope (*B* = 0.023 per 1° increment, *p* < 0.0001, figure 3*b*). There was also an interaction effect (*p* = 0.015, figure 3*b*), where compared to level ground, UL Sol iEMG increased on steeper uphill slopes (+6° and +9°) for both prostheses (*p* < 0.008), and was lower while using the BiOM compared to the ESAR prosthesis on +9° (*p* < 0.001). TA iEMG increased for each steeper uphill slope and decreased for each steeper downhill slope (*B* = 0.006–0.014 per 1° increment, *p* < 0.001, figure 3*c*). There was also an interaction effect (*p* = 0.046, figure 3*c*), where compared to level ground, TA iEMG increased on +9° while using the ESAR prosthesis (*p* < 0.001) and use of the BiOM compared to the ESAR prosthesis lowered TA iEMG on +9° (*p* = 0.009).

### 3.2. Unaffected leg peak electromyography

**Figure 4.**
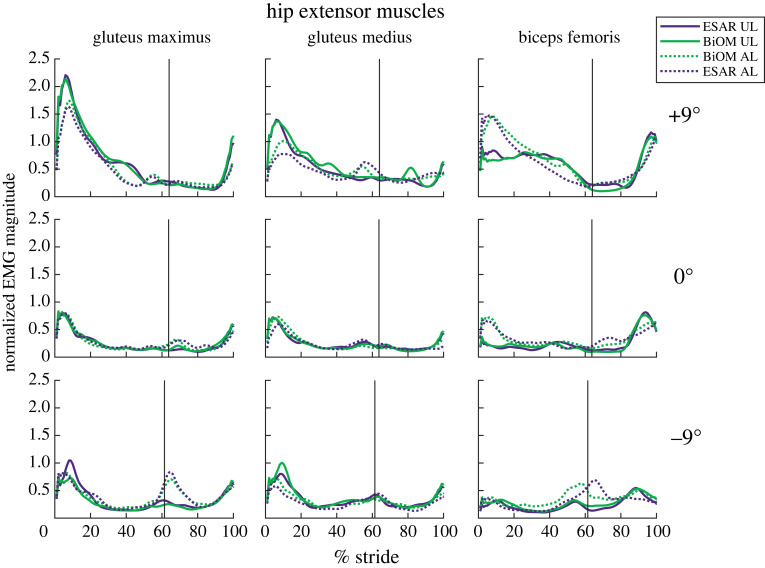
Average magnitude of muscle activity normalized to peak EMG while using the ESAR prosthesis during walking on level ground for the unaffected leg (UL—solid line) and affected leg (AL—dotted line) gluteus maximus, gluteus medius and biceps femoris muscles while subjects walked at 1.25 m s^−1^ using their own elastic storage and return (ESAR—purple) prosthesis and the BiOM (green) powered prosthesis on level ground and ±9° slopes. The black vertical line represents average percentage of a stride where toe-off occurred for each slope.

For the UL hip extensors, Gmax, Gmed and BF peak EMG increased for each steeper uphill slope (*B* = 0.05–0.16 per 1° increment, *p* < 0.001, figure 4), Gmed peak EMG increased for each steeper downhill slope (*B* = −0.01 per 1° increment, *p* = 0.046, figure 4) and BF peak EMG decreased for each steeper downhill slope (*B* = 0.025 per 1° increment, *p* = 0.001, figure 4).

**Figure 5.**
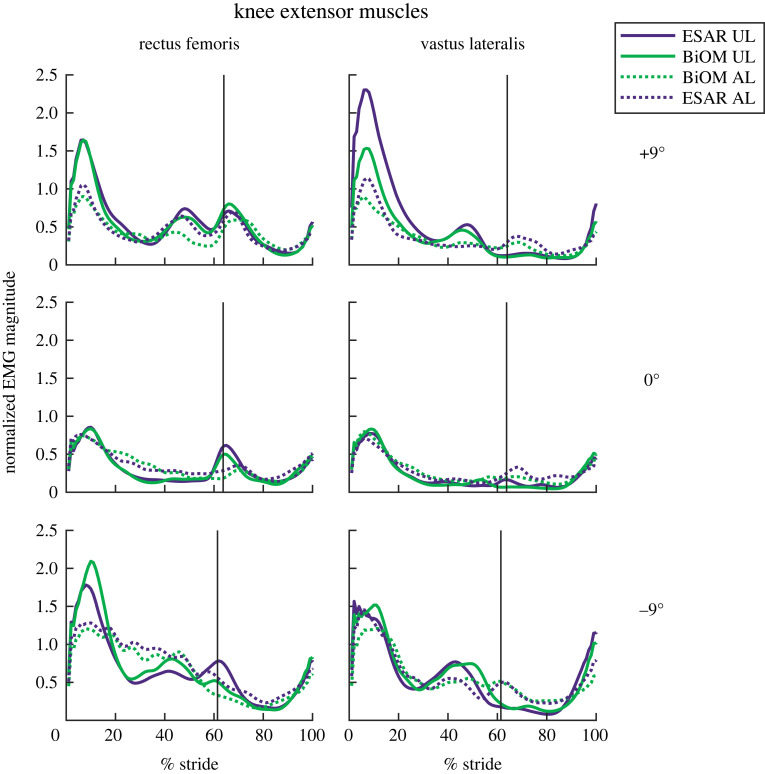
Average magnitude of muscle activity normalized to peak EMG while using the ESAR prosthesis during walking on level ground for the unaffected leg (UL—solid line) and affected leg (AL—dotted line) rectus femoris and vastus lateralis muscles while subjects walked at 1.25 m s^−1^ using their own elastic storage and return (ESAR—purple) prosthesis and the BiOM (green) powered prosthesis on level ground and ±9° slopes. The black vertical line represents average percentage of a stride where toe-off occurred for each slope.

For the UL knee extensors, RF and VL peak EMG increased for each steeper uphill (*B* = 0.079–0.088 per 1° increment, *p* < 0.001, figure 5) and downhill (*B* = −0.131 to −0.082 per 1° increment, *p* < 0.001, figure 5) slope.

**Figure 6.**
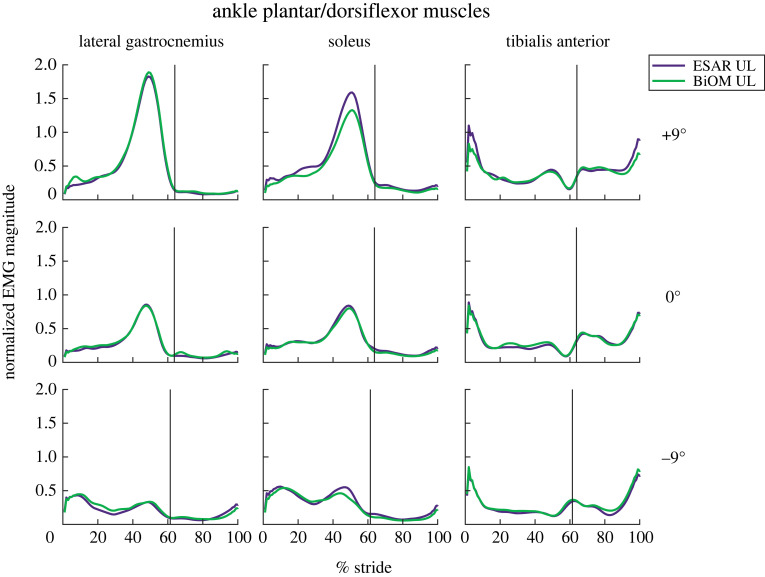
Average magnitude of muscle activity normalized to peak EMG while using the ESAR prosthesis during walking on level ground for the unaffected leg (UL) lateral gastrocnemius, soleus and tibialis anterior muscles while subjects walked at 1.25 m s^−1^ using their own elastic storage and return (ESAR—purple) prosthesis and the BiOM (green) powered prosthesis on level ground and ±9° slopes. The black vertical line represents average % stride in which toe-off occurred for each slope.

For the UL plantar- and dorsiflexors, LG peak EMG increased for each steeper uphill slope (*B* = 0.12 per 1° increment, *p* < 0.001, figure 6) and decreased for each steeper downhill slope (*B* = 0.04 per 1° increment, *p* < 0.01, figure 6). Sol peak EMG increased with each steeper uphill slope (*B* = 0.096 per 1° increment, *p* < 0.001, figure 6), decreased with each steeper downhill slope (*B* = 0.02 per 1° increment, *p* = 0.002, figure 6) and was higher when using the BiOM compared to an ESAR prosthesis on downhill slopes (*B* = 0.12, *p* = 0.0127, figure 6). TA peak EMG increased with each steeper uphill slope (*B* = 0.032 per 1° increment, *p* < 0.002, figure 6). There was also an interaction effect (*p* = 0.018, figure 6), where compared to level ground, when using the ESAR prosthesis, TA peak EMG on +9° was greater than on level ground (*p* = 0.012).

### 3.3. Unaffected leg muscle activity burst duration

**Figure 7.**
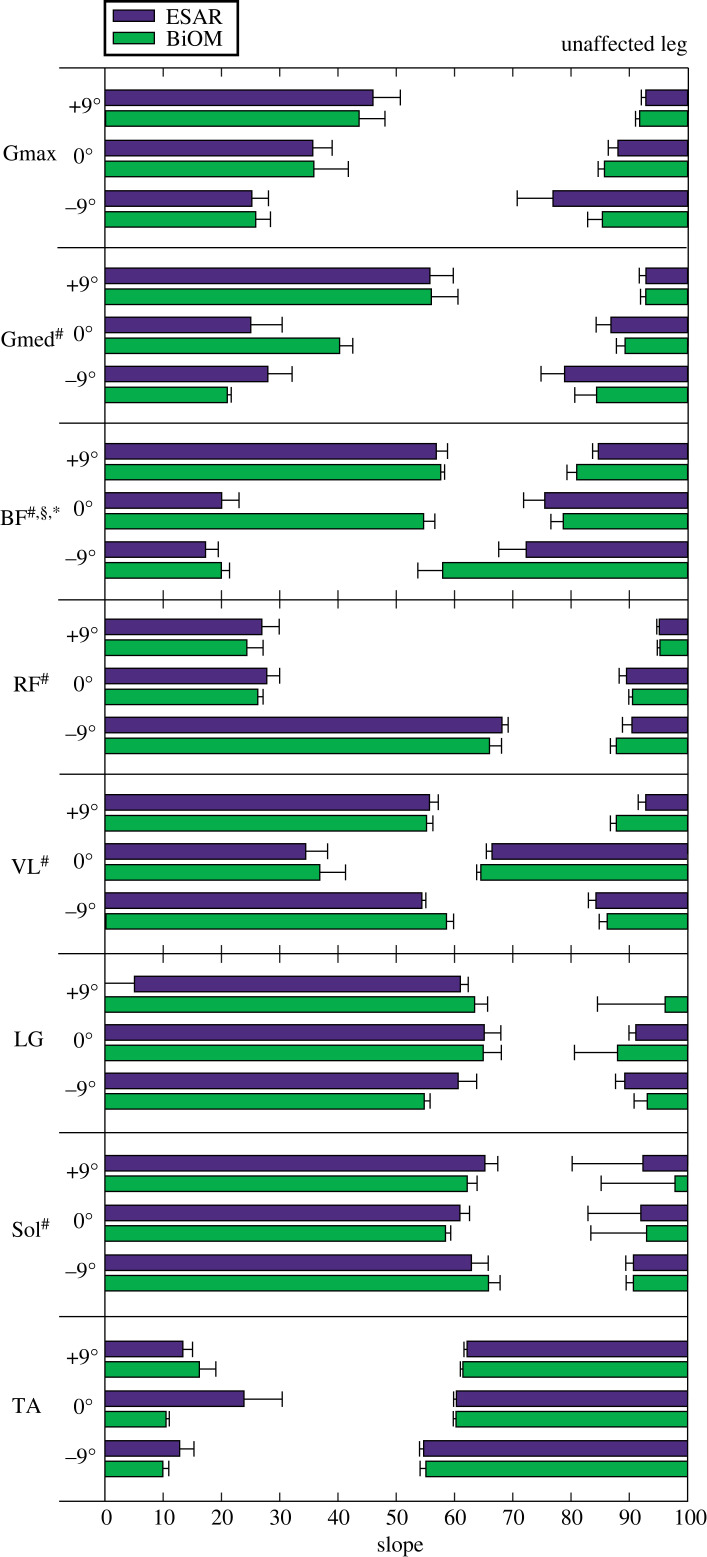
Average (±s.e.m.) duration of the primary burst of muscle activity for the unaffected leg gluteus maximus (Gmax), gluteus medius (Gmed), biceps femoris (BF), rectus femoris (RF), vastus lateralis (VL), lateral gastrocnemius (LG), tibialis anterior (TA) and soleus (Sol) muscles while subjects walked at 1.25 m s^−1^ using their own elastic storage and return (ESAR—purple) prosthesis and the BiOM (green) powered prosthesis on level ground and ±9° slopes. Hash symbol (#) indicates a significant main effect of slope. Section symbol (§) indicates a significant main effect of prosthesis type. Asterisk (*) indicates significant interaction effect between prosthesis and slope. *α* = 0.05.

For the UL hip extensors, Gmed burst duration was longer for each steeper uphill slope (*B* = 1.44 per 1° increment, *p* = 0.047, figure 7). BF duration was longer with each steeper uphill slope (*B* = 3.07 per 1° increment, *p* < 0.001, figure 7), and when using the BiOM compared to the ESAR prosthesis during uphill and downhill walking (*B* = 13.8–18.2, *p* < 0.001, figure 7). There was also an interaction effect (*p* = 0.001, figure 7), where compared to level ground, BF burst duration using the ESAR prosthesis was longer on all uphill slopes (*p* < 0.001), and BF burst duration using the BiOM prosthesis was longer on level ground (*p* < 0.0001) compared to the ESAR prosthesis.

For the UL knee extensors, RF burst duration was longer when using the BiOM compared to the ESAR prosthesis on uphill slopes (*B* = 7.66 per 1° increment, *p* = 0.041, figure 7), and was longer for each steeper downhill slope (*B* = −5.20 per 1° increment, *p* < 0.0001, figure 7). VL burst duration was longer for each steeper uphill (*B* = 1.23 per 1° increment, *p* = 0.002, figure 7) and downhill (*B* = −2.49 per 1° increment, *p* < 0.001, figure 7) slope.

For the UL plantar- and dorsiflexors, LG burst duration was shorter for each steeper uphill (*B* = −1.34 per 1° increment, *p* < 0.001, figure 7) and downhill (*B* = 0.61 per 1° increment, *p* = 0.018, figure 7) slope. Sol burst duration was longer for each steeper uphill slope (*B* = −0.712 per 1° increment, *p* = 0.004, figure 7).

### 3.4. Affected leg integrated electromyography

For the AL hip extensors, Gmax and Gmed iEMG increased for each steeper uphill slope (*B* = 0.22–0.23 per 1° increment, *p* < 0.001, figure 1), and Gmax iEMG increased for each steeper downhill slope (*B* = −0.007 per 1° increment, *p* < 0.003, figure 1*b*). There was also an interaction effect (*p* = 0.033, figure 1*b*), where Gmax iEMG increased on steeper uphill slopes (+6° and +9°) compared to level ground (*p* < 0.003). BF iEMG increased for each steeper uphill slope (*B* = 0.021 per 1° increment, *p* < 0.001, figure 1*f*). There was also an interaction effect (*p* = 0.015, figure 1*f*), where BF iEMG increased for each steeper uphill slope compared to level ground (*p* = 0.003–0.0297), and use of the BiOM compared to the ESAR prosthesis increased BF iEMG on +6° and +9° (*p* = 0.007–0.0078).

For the AL knee extensors, RF iEMG increased for each steeper uphill (*B* = 0.011 per 1° increment, *p* < 0.001, figure 2*b*) and downhill (*B* = −0.033 per 1° increment, *p* < 0.001, figure 2*b*) slope. VL iEMG increased for each steeper downhill slope (*B* = −0.025, *p* < 0.001, figure 2*d*).

### 3.5. Affected leg peak electromyography

For the AL hip extensors, Gmax, Gmed and BF peak EMG increased for each steeper uphill slope (*B* = 0.065–0.117 per 1° increment, *p* < 0.001, figure 4), Gmax peak EMG increased (*B* = −0.018 per 1° increment, *p* = 0.048, figure 4) for each steeper downhill slope, and BF peak EMG decreased (*B* = 0.02 per 1° increment, *p* = 0.024, figure 4) for each steeper downhill slope.

For the AL knee extensors, RF and VL peak EMG increased for each steeper uphill (*B* = 0.024–0.065 per 1° increment, *p* < 0.05, figure 5) and downhill (*B* = −0.12 to −0.084 per 1° increment, *p* < 0.001, figure 5) slope.

### 3.6. Affected leg muscle activity burst duration

**Figure 8.**
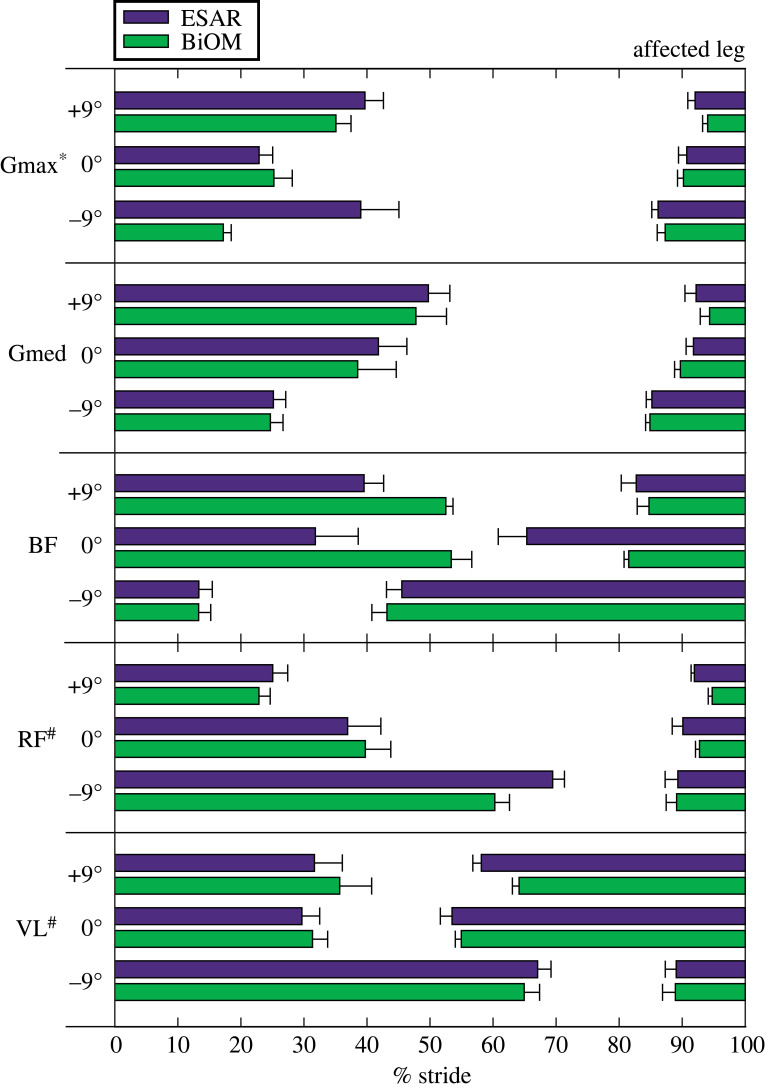
Average (±s.e.m.) duration of primary burst of muscle activity for the affected leg gluteus maximus (Gmax), gluteus medius (Gmed), biceps femoris (BF), rectus femoris (RF) and vastus lateralis (VL) muscles while subjects walked at 1.25 m s^−1^ using their own elastic storage and return (ESAR—purple) prosthesis and the BiOM (green) powered prosthesis on level ground and ±9° slopes. Hash symbol (#) indicates a significant main effect of slope. Asterisk (*) indicates significant interaction effect between prosthesis and slope. *α* = 0.05.

For the AL hip extensors, Gmax burst duration increased with each steeper uphill (*B* = 0.72 per 1° increment, *p* = 0.032, figure 8) and downhill (*B* = −1.41 per 1° increment, *p* = 0.014, figure 8) slope. There was also an interaction effect (*p* = 0.034, figure 8), where Gmax burst duration was longer when using the ESAR compared to BiOM prosthesis on a −9° slope (*p* = 0.009). BF burst duration was longer when using the BiOM compared to the ESAR prosthesis on uphill slopes (*B* = 7.10, *p* = 0.006, figure 8).

For the AL knee extensors, RF burst duration decreased for each steeper uphill slope (*B* = −1.13 per 1° increment, *p* = 0.01, figure 8) and increased for each steeper downhill slope (*B* = −3.17, *p* < 0.001, figure 8), and VL burst duration increased for each steeper downhill slope (*B* = −4.83, *p* < 0.001, figure 8).

## 4. Discussion

We compared the leg muscle activity of people with TTA during walking on level ground, uphill and downhill slopes while they used a passive-elastic compared to powered ankle–foot prosthesis. Differences in leg muscle activity could elucidate differences in the metabolic cost of walking while using a passive-elastic versus powered ankle–foot prosthesis. Based on our results, we partially accept our first hypothesis that iEMG and peak EMG magnitude of the hip extensors and knee extensors of each leg would decrease, and muscle activity burst duration would not differ during uphill and level-ground walking when subjects with TTA used the BiOM compared with an ESAR prosthesis. We also partially accept our second hypothesis that iEMG, peak EMG magnitude and muscle activity burst duration of all leg muscles for the AL and UL would not differ during downhill walking when subjects with TTA used the BiOM compared with an ESAR prosthesis. When subjects with TTA used the BiOM compared with an ESAR prosthesis, UL lateral gastrocnemius iEMG increased on downhill slopes and AL biceps femoris iEMG increased on +6° and +9° slopes, and UL rectus femoris iEMG decreased on uphill slopes, UL vastus lateralis iEMG decreased on +6° and +9° slopes, and UL soleus and tibialis anterior iEMG decreased on a +9° slope. In addition, use of the BiOM compared to an ESAR prosthesis increased Sol peak EMG on downhill slopes, increased AL and UL BF and UL RF burst duration on uphill slopes, and increased UL BF burst duration on downhill slopes. Thus, use of the BiOM compared to each subject's ESAR prosthesis did not systematically decrease iEMG, peak EMG or muscle activity burst duration on uphill slopes and had some effects on downhill slopes.

To walk uphill compared to level ground, non-amputees and people with TTA increase leg extensor muscle activity to increase the positive mechanical work produced by the legs and raise the COM [10,20–22]. To walk downhill compared to level ground, non-amputees and people with TTA increase knee extensor muscle activity to increase the magnitude of negative mechanical work absorbed by the legs and lower the COM [20–22]. When people with TTA walked on level, uphill and downhill slopes, they exhibited the same changes in muscle activity patterns as non-amputees [10]. Specifically, similar to non-amputees, during walking on uphill slopes, people with TTA using the BiOM or an ESAR prosthesis increased activity of the hip extensor, knee extensor and ankle plantar- and dorsiflexor muscles, and during walking on downhill slopes, increased activity of the knee extensor muscles compared to level ground. In addition, for each increase in slope from 0° to +9°, peak EMG increased for the hip extensors, knee extensors and ankle dorsi- and plantar-flexors and for each decrease in slope from 0° to −9°, peak EMG decreased for the hip extensors, increased for the knee extensors and decreased for the ankle dorsi- and plantar-flexors. Moreover, for each change in slope from 0° to ±9°, we found that people with TTA using either prosthesis had longer hip and knee extensor, and no change in ankle dorsi- and plantar-flexor burst duration over a stride. Our findings agree with previous studies which found that non-amputees increased hip extensor muscle activity amplitude and burst duration on a +39% grade (+21.3° slope), and knee extensor muscle activity amplitude on a −39% grade (−21.3° slope), and ankle dorsi- and plantar-flexor muscle activity amplitude on a +39% grade compared to walking on level ground at a self-selected speed [10].

Though the BiOM prosthesis provides stance phase power and reduces muscle activity in some of the leg muscles during walking, it has greater mass compared to an ESAR prosthesis, which may require increased leg muscle activity. When walking on +6° and +9° slopes, people with TTA using the BiOM compared to an ESAR prosthesis increased AL BF iEMG by 21–26%. Additionally, we detected a significantly longer AL BF burst duration on uphill slopes but did not find any differences in peak EMG between prostheses. The duration of UL BF was significantly longer when people with TTA used the BiOM compared to the ESAR to walk on uphill and downhill slopes (figure 7). The difference in duration is predominantly due to an earlier deactivation during the stride when using the ESAR compared to the BiOM. The earlier deactivation point when using the ESAR compared to the BiOM is likely a result of greater step-to-step variation between strides when using the ESAR prosthesis, contributing to a higher activation threshold and earlier detection of muscle deactivation during a stride. Moreover, we found no differences in iEMG for the AL uni-articular hip extensors (Gmax and Gmed) between prostheses. Thus, the increase in AL iEMG for the bi-articular BF may be due to differences in the knee flexion torque required. It is possible that the increased mass of the BiOM (2300 g) compared to the ESAR (574 g) prosthesis required greater AL BF iEMG for knee flexion so that the prosthesis would clear the ground during swing phase and the lower leg would slow prior to ground contact. A previous study found that adding mass to an ESAR prosthesis so that the estimated UL and AL mass of people with TTA was equal (added 2360 g to the AL, on average) increased the metabolic cost of level-ground walking at 1.2 m s^−1^ by 12% [23]. Thus based on this previous study, the additional 1726 g of the BiOM compared to an ESAR prosthesis could account for the increased BF iEMG and would increase metabolic cost by approximately 9% during level-ground walking. However, people with TTA using the BiOM compared to an ESAR prosthesis had an 8% lower metabolic cost during level-ground walking at 1.25 m s^−1^ [6]. Thus, it is likely that there are other muscle activity differences between people with TTA using the BiOM compared to a passive-elastic prosthesis to walk on level ground, such as lower UL RF activity (which we observed in this study), or lower trunk muscle activity. Moreover, future research is needed to determine how the mass of and power provided by the BiOM prosthesis affect metabolic costs and specifically BF muscle activity in the AL.

Increased trailing AL prosthetic step-to-step transition work when using the BiOM compared to a passive-elastic prosthesis likely lowers the magnitude of mechanical work and muscle activity in the leading UL. When subjects with TTA used the BiOM compared to their own ESAR prosthesis, they decreased UL knee extensor iEMG during walking on uphill slopes. Specifically, use of the BiOM versus ESAR prosthesis decreased UL RF iEMG by 8–12% on uphill slopes, and VL iEMG by 22–39% on +6° and +9° slopes. A previous study by Bateni and Olney found that people with TTA using a passive prosthetic foot (SAFE—stationary attachment flexible endoskeleton) during level-ground walking at a self-selected walking speed had greater UL knee flexion angles during early stance compared to non-amputees. Bateni & Olney [24] inferred that because the SAFE prosthesis could not actively plantarflex at the end of the stance phase, the user's initial COM position was lower during the step-to-step transition from the trailing AL to the leading UL compared to the COM position of non-amputees during the step-to step transition, which would result in a more flexed knee. Additionally, they inferred that a more flexed UL knee enabled subjects to produce more propulsive force from knee extension in late stance compared to non-amputees to compensate for inadequate prosthetic ankle push-off work. When walking at 1.24 m s^−1^ on level ground, use of the BiOM compared to an ESAR prosthesis results in 63% greater net positive trailing AL step-to-step transition work, and during walking on a +5° slope at 1.24 m s^−1^, results in 53% greater net positive trailing AL step-to-step transition work [7]. Thus, the combination of powered prosthetic plantar-flexion and greater positive trailing AL step-to-step transition work when using the BiOM compared to a passive-elastic prosthesis to walk uphill likely results in a more extended UL knee angle during stance, and reduces the demand on the UL knee extensors to support bodyweight and produce work during a step.

Increased positive prosthetic work when using the BiOM compared to an ESAR prosthesis to walk downhill may influence the ankle joint work of the UL. When subjects with TTA used the BiOM compared to their own ESAR prosthesis, they changed UL plantar-flexor iEMG during walking on downhill slopes. Specifically, use of the BiOM versus ESAR prosthesis increased LG iEMG by 6–8%, and did not change peak EMG or burst duration. To maintain constant velocity when walking downhill, net negative work must be performed on the COM. When walking at 1.25 m s^−1^ on downhill slopes using an ESAR prosthesis, the UL and AL ankle of people with TTA performs net negative work over a stride [25]. However, when using the BiOM to walk at the same speed on the same downhill slope, net prosthetic ankle work of the AL is significantly more positive compared to when using an ESAR prosthesis [25]. Thus, to maintain constant downhill walking velocity using the BiOM, the UL must perform greater net negative work compared to when using an ESAR prosthesis, which may be accomplished through greater activation of the UL LG.

Greater positive prosthetic work when using the BiOM compared to an ESAR prosthesis likely reduces the amount of mechanical work and muscle activity needed from UL plantar-flexor muscles to walk uphill. When subjects with TTA used the BiOM compared to their own ESAR prosthesis, Sol and TA iEMG were not different during walking on downhill slopes, but were 21–26% lower during walking on the +9° slope. When people with TTA walk at 1.25 m s^−1^ on +6° and +9° slopes using the BiOM compared to an ESAR prosthesis, they exhibit 89% and 55% greater positive prosthetic ankle work, respectively [25]. This positive prosthetic ankle work results in greater trailing AL net positive step-to-step transition work. When walking at 1.24 m s^−1^ on level ground, the trailing AL using the BiOM provides 63% greater net positive step-to-step transition work compared to an ESAR prosthesis, and during walking on a +5° slope at 1.24 m s^−1^, provides 53% greater net positive step-to-step transition work compared to an ESAR prosthesis [7]. The role of the gastrocnemius and soleus in relation to forward propulsion is still unclear, but Franz *et al*. concluded the horizontal propulsive force that facilitates trailing leg step-to-step transition work is primarily generated by the gastrocnemius, with the soleus contributing to propulsion when the demand is higher [26–28]. When walking uphill, the parallel propulsive force demand increases compared to level ground, and both legs contribute positive work to raise the COM up the slope [22]. Greater (more positive) prosthetic ankle net mechanical work over a stride during walking using the BiOM compared to an ESAR prosthesis likely reduces the propulsive demand and amount of positive net mechanical work that the trailing UL must produce to maintain a constant walking speed, which in turn could result in lower UL Sol iEMG. Future research is needed to determine how the power output magnitude of the BiOM affects individual leg step-to-step transition work and gastrocnemius and soleus iEMG during walking on level ground and slopes. Moreover, the TA often co-activates with the Sol during ground contact and acts to stabilize the ankle [29]. It is possible that the power provided by the BiOM allows users to reduce UL co-contraction during walking on uphill slopes compared to an ESAR prosthesis and could explain why TA iEMG was lower during walking on a +9° slope, but not on level ground or on less steep uphill slopes (+3° and +6°). While using an ESAR or the BiOM prosthesis to walk on downhill slopes, the UL ankle absorbs more negative work than the AL prosthetic ankle but there is no effect of prosthetic foot type on UL ankle negative or net mechanical work [25]. Thus, people with TTA would not likely exhibit differences in Sol or TA iEMG when using the BiOM to walk on downhill slopes.

Previous studies found that metabolic power was lower in people with TTA using the BiOM compared to an ESAR prosthesis during level-ground walking at speeds of 0.75–1.75 m s^−1^ and on +3° and +6° slopes at 1.25 m s^−1^. We found that use of the BiOM compared to an ESAR prosthesis resulted in 6–8% greater UL LG iEMG on downhill slopes, and 21–26% greater AL BF iEMG on +6° and +9° slopes, and 8–12% lower UL RF iEMG on uphill slopes, and 21–39% lower UL Sol, TA, and VL on +6° and +9° slopes. These differences in iEMG between prostheses do not provide a clear mechanism for differences in metabolic power. Moreover, previous studies have inferred that more proximal muscles, such as those surrounding the hip joint, may be less economical than more distal muscles due to different inter-muscular muscle–tendon architecture [30], which further complicates the inference between muscle activity and metabolic power. Though iEMG characterizes muscle activity magnitude and duration, and is associated with the development of muscle force, which incurs a metabolic cost, differences in leg muscle iEMG when using a passive-elastic versus stance-phase powered prosthesis do not directly relate with differences in metabolic power when using these prostheses. Musculoskeletal models that use EMG can well predict metabolic cost, but require additional variables such as muscle force, muscle efficiency, joint angles and moments and muscle shortening and lengthening velocities [31]. Thus, future research using musculoskeletal models of people with TTA using an ESAR and BiOM prosthesis may provide more insight into how changes in proximal and distal muscle iEMG contribute to the overall metabolic cost of walking on level ground and slopes.

There were some potential limitations that may have affected the results of our study. The subjects in the present study were on average 19.8 kg lighter than subjects in Herr & Grabowski [6], and 13.5 kg lighter than subjects in Kim *et al*. [9] when wearing their own ESAR prosthesis. Thus, the mass of the BiOM comprised a greater proportion of body mass and may have required greater BF iEMG in the present study, which could contribute to a greater metabolic cost during walking on level ground compared to Herr & Grabowski [6]. Additionally, we tuned the BiOM to match non-amputee ankle angle range of motion, peak moment, net mechanical work and peak power at each slope, and these settings were kept constant throughout the study [25]. As subjects accommodated to using the BiOM, it is possible that the tuning settings resulted in prosthetic ankle biomechanics that were no longer within 2 s.d. from non-amputee average values [8]. If the change in user response to the BiOM resulted in prosthetic ankle biomechanics that were different from non-amputee values, it is possible that muscle activation changed in the AL and UL to compensate. Further research is needed to determine the long-term effects of tuning parameters and the use of a powered prosthesis on biomechanics, metabolic costs and leg muscle activity during walking.

## 5. Conclusion

We found that people with TTA using a stance-phase battery-powered prosthesis (BiOM) compared to an ESAR prosthesis have 21–26% greater AL BF iEMG, and 8–39% lower UL VL, RF, Sol and TA iEMG on uphill slopes, and 6–8% greater LG iEMG on downhill slopes during walking at 1.25 m s^−1^. Regardless of the prosthesis used, hip, knee and ankle extensor muscle iEMG increased with each steeper uphill slope, whereas hip and knee extensor iEMG increased and ankle extensor iEMG decreased with each steeper downhill slope. Our results may be used to inform future prosthetic design. For example, lower prosthetic mass may lessen the muscle activity of the AL during walking. Additionally, changes in iEMG have been associated with changes in metabolic power. Thus, additional research is needed to determine how the mass and power output of a powered ankle–foot prosthesis affect iEMG and the metabolic cost of people with TTA during walking on level ground and slopes.

## Data Availability

The authors confirm that all data underlying the findings are fully available without restriction: https://doi.org/10.6084/m9.figshare.19694737.v4 [32]. All relevant data are within the paper.
